# Measurement and Analysis of Central Corneal Thickness at Different Postnatal Stages in Chinese Premature Infants

**DOI:** 10.1155/2020/7313909

**Published:** 2020-09-16

**Authors:** Chenyi Liu, Hanfei Wu, Jimeng Lao, Sulan Wu, Xiaoqiong Xu, Zhe Lv, Jianbo Mao

**Affiliations:** ^1^Chicago College of Optometry, Midwestern University, Downers Grove, IL, USA; ^2^The People's Hospital of Zhuji, Shaoxing, Zhejiang Province, China; ^3^Affiliated Eye Hospital of Wenzhou Medical University, Hangzhou, Zhejiang Province, China

## Abstract

**Purpose:**

The objective of this study was to evaluate central corneal thickness (CCT) in Chinese premature infants at different postnatal stages to study the peak point and analyze influential factors on CCT development.

**Methods:**

This was a cross-sectional study of premature infants. Initial CCT measurement was taken at 34 weeks of gestational age (GA) and at intervals until 88 weeks of postmenstrual age (PMA) was reached. The comparison and correlation analysis were carried out to access the association of CCT with gender, birth weight (BW), GA, and retinopathy of prematurity (ROP) for each PMA. The premature infants were divided into the thick CCT group and the thin CCT group according to the average CCT at 40 w. And the difference in CCT between the two groups at subsequent 52 w and 64 w was compared.

**Results:**

A total of 1726 premature infants (3463 measurements) with an average of 2.21 ± 1.57 measurements were included in this study. The CCT decreased from 34 w GA to 52 w PMA (*R* = 92.36, *P* < 0.0001) and then reached a plateau (*R* = 2.541, *P*=0.3567). Male (*P* < 0.05), low BW (*P* < 0.05), and low GA (*P* < 0.05) were associated with thicker CCT at the early stage of PMA. The premature infants who had thick CCT at 40 w would have thick CCT at 52 w and 64 w accordingly.

**Conclusions:**

The CCT values of premature infants decreased over time and plateaued at 52 w PMA. Gender, BW, and GA were considered as the influential factors of CCT at the early stage of PMA. Moreover, CCT at 40 w could forecast its development trend at 52 w or 64 w after birth.

## 1. Introduction

Central corneal thickness (CCT) has become an important aspect of diagnosing and treating various eye disorders [1]. There are many techniques for measuring CCT, such as ultrasonic pachymetry, Visante optical coherence tomography, and Fourier-domain optical coherence tomography [2]. The accurate measurement of corneal thickness is vital to evaluate the endothelial function and to manage pediatric glaucoma [3]. Lope et al. demonstrated in their study that the mean CCT was higher in the pediatric glaucoma group [4]. Rebecca et al. also showed that CCT was a phenotypic risk factor for primary open angle glaucoma (POAG) in a genomic study [5].

In general, premature infants were born with immature organs and tissues in their physiological conditions. CCT in premature infants and full-term newborns is significantly thicker compared to adult corneal thickness [6]. Among infants, CCT in premature infants was also found to be thicker than in full terms [7–9]. Few studies discussed the CCT development in premature infants during the neonatal period. Choo et al. examined the premature infants at 2 different points, reported a significant decrease in CCT between 1^st^ at “premature” (PMA between 32 and 36 weeks) and 2^nd^ at “term” (PMA between 37 and 41 weeks) [6]. Another study also suggested a decrease in CCT from 794 to 559 *µ*m between 24 and 32 w of GA in 56 premature infants [10].

In addition, researches also showed that CCT in premature infants was negatively correlated with GA and birth weight (BW) at early phases, which indicated that premature infants with higher GA and BW would have lower CCT values [11, 12]. This agreed to the decrease in CCT with the growth of infants. Overall, however, these studies had a shorter follow-up period and a relatively small sample size. They also had not discussed whether GA, BW, and presence of retinopathy of prematurity (ROP) were potential influential factors in change in CCT.

Our study aimed to analyze the dynamic change in CCT in a large sample of premature infants within a period of 1 year and to explore whether gender, GA, BW, and presence of ROP could be influential factors in CCT changes.

## 2. Patients and Methods

This study was a cross-sectional clinical trial conducted on premature infants at the Affiliated Eye Hospital of Wenzhou Medical University (Hangzhou, China), from February 2014 to April 2018. It followed the tenets of the Declaration of Helsinki. Local ethical approval was obtained from the ethics committee of the Affiliated Eye Hospital of Wenzhou Medical University, Zhejiang, China. Informed consent was obtained from all premature infants' parents. A total of 1726 premature infants were screened for ROP and recruited in this study. The age subjects were screened at 34 w, 36 w, 38 w, 40 w, 42 w, 44 w, 46 w, and 48 w GA and 52 w, 64 w, 76 w, and 88 w postmenstrual age (PMA). Infants with neurological anomalies, systemic syndromes, and other ocular disease such as congenital keratopathy and congenital microphthalmos syndromes were excluded. Gender, BW, GA, PMA, and presence of ROP were recorded at each visit. The follow-up time was 1 year in total.

Prior to ROP screening, the eyelids of infants were opened with a pediatric speculum, followed by instillation of proparacaine hydrochloride 0.5% (Alcaine-Alcon, USA). Then, the CCT measurements were performed using hand-held corneal pachymeter (PachPen, Accutome, USA). Prior to testing, the probe tip was disinfected using an alcohol swab and was then placed at the central corneal surface gently after complete evaporation of alcohol. Three measurements were taken on each eye using supine position. The average reading was used for analysis. Prophylactic ofloxacin eye ointment was prescribed two times a day in both eyes for three days after the procedure. All operations were completed by one experienced ophthalmologist (Dr. Mao). None of the infants had any sign of adverse effects before, during, or after the procedure.

Statistical analysis was performed using SPSS version 22 (IBM Corp., Armonk, NY, USA). There was no statistically significant difference between the two eyes. Thus, the data of the right eye were used for analysis. Mean CCT was expressed in mean ± standard error (SE). Other parameters were recorded as mean ± deviation (SD). Independent-sample *t*-test was used to study association between CCT and gender, BW, GA, and ROP subgroups. One-way ANOVA was used to analyze the CCT at 40 w, 52 w, and 64 w in the thin CCT group and thick CCT group, respectively. A value of *P* < 0.05 was set for statistical significance.

## 3. Results

**Figure 1:**
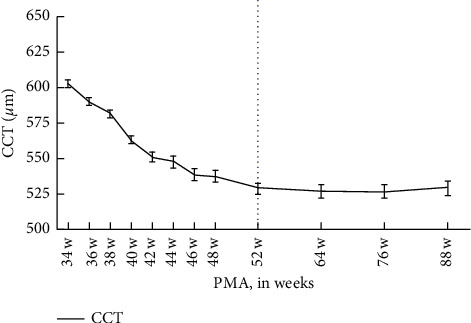
Trend of CCT. PAM: postmenstrual age; CCT: central corneal thickness. Correlation between PMA and CCT before 52 w: *y* = −4.241*x* + 739.5; correlation between PMA and CCT after 52 w: *y* = 0.08250*x* + 521.6.

A total of 1726 premature newborns (3463 measurements) were included in this study. Subjects were evaluated longitudinally 1 to 7 times (in total 3463 times). 943 were male, and 783 were female. The mean BW was 1556.40 ± 409.63 g (range: 670–3790 g). The mean GA was 30.99 ± 2.22 w (range: 23–39 w) (Table 1). The mean CCT of premature infants at 34 w was 602.67 ± 57.26 *μ*m, which gradually declined with the increase in PMA. At 52 w of PMA, the mean CCT reached 529.17 ± 38.70 *μ*m (*R* = 92.36, *P* < 0.0001) and slowly plateaued at 530 *μ*m (*R* = 2.541, *P*=0.3567) (Table 2 and Figure 1).

**Figure 2:**
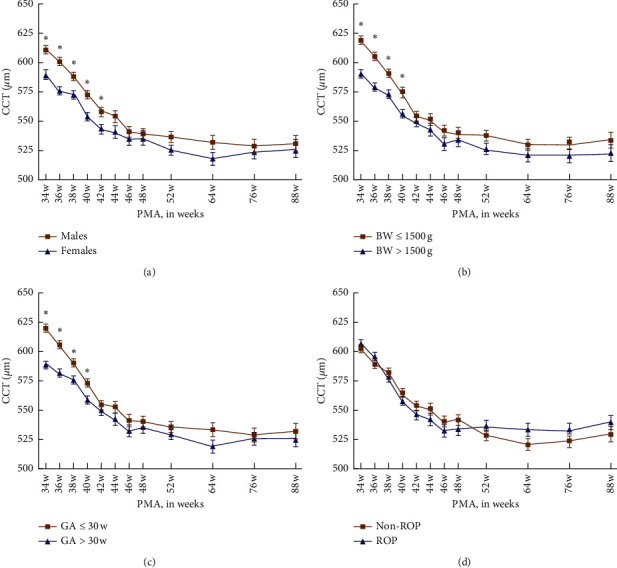
(a) Mean CCT of different genders at each week of PMA; (b) mean CCT of different BWs at each PMA; (c) mean CCT of different GAs at each PMA; (d) mean CCT in the ROP subgroup and without the ROP subgroup at different PMAs. ^*∗*^*P* < 0.05.

The mean CCT of males was consistently higher than that of females during the early period (≤42 w) (all *P* < 0.05). After 42 w, there was no significant difference between males and females at any PMA (Table 3 and Figure 2(a)).

The mean CCT of low BW (<1500 g) was consistently thicker than that of the high BW (>1500 g) during the early period (≤40 w) (all *P* < 0.05). After 40 w, there was no significant difference between low and high BW at any PMA (Table 4 and Figure 2(b)).

The mean CCT of low GA (<30 w) was consistently thicker than that of the high GA (>30 w) during the early period (≤40 w) (all *P* < 0.05). After 40 w, there was no significant difference between low and high GA at any PMA (Table 5 and Figure 2(c)).

There was no significant difference between groups with and without ROP at any PMA (Table 6 and Figure 2(d)).

**Figure 3:**
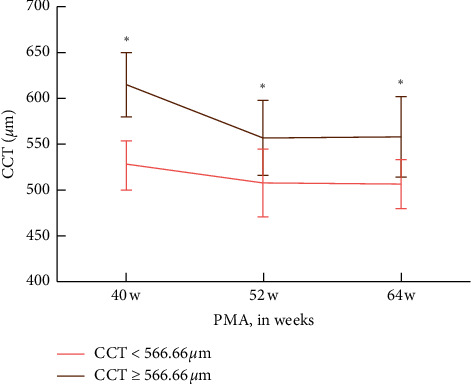
CCT variation trend at 40 w, 52 w, and 64 w. ^*∗*^*P* < 0.05.

At 40 w, the average CCT was 566.66 ± 53.00 *µ*m in 65 eyes from 65 premature infants. We divided these infants into two groups, with 36 having thin CCT < 566.66 *µ*m and 29 eyes having thick CCT ≥ 566.66 *µ*m. Keep a record of CCT in each infant at 40 w, 52 w, and 64 w. The results showed that two groups both kept a decline all the time (*P*=0.005 in the thin CCT group and *P* < 0.001 in the thick CCT group). Furthermore, CCT change was significantly different between the two groups at 40 w (*t* = −11.50, *P* < 0.001), 52 w (*t* = −4.98, *P* < 0.001), and 64 w (*t* = −5.86, *P* < 0.001) (Table 7 and Figure 3).

## 4. Discussion

Both central and peripheral corneal thickness had been studied in adults in several researches [13, 14]; however, very little was known about the CCT in premature infants. We studied the trend of CCT change in premature during the 1-year period using a large sample size. This study was so far the largest and longest study on the CCT in premature infants. We found that, at early stage of PMA (before 52 w), the CCT of premature infants decreased gradually from 602.67 ± 57.26 *µ*m to 529.17 ± 38.70 *µ*m and then stabilized from 52 w to one year at approximately 530 *μ*m.

Kirwan demonstrated that CCT in premature infants decreased from 691 *µ*m at 31 w to 605 *µ*m at term [15]. Studies by Jethani et al. had showed the CCT of preterm infants(<260 days) decreased from 620.7 ± 88.8 *µ*m to 534.1 ± 57.6 *µ*m at the end of one year [16]. Our finding at the early stage of PMA was consistent with these studies. With the longer observation period in our study, we were able to analyze changes in CCT up to 1 year and map out the trend of change more precisely with a larger sample base. In our study, we discovered that the CCT did not continuously decrease until term. The speed of change decreased at later PMA, and the CCT stabilized after.

In Rushood study, the CCT of males was significantly higher than females [17]. In our study, the curve in Figure 2 showed that the CCT of males was slightly thicker than females in the early period. Although CCT at each PMA was higher in males, a statistically significant difference was only found prior to 42 weeks.

Uva et al. found a significant correlation between CCT and BW [12]. Gunay observed a negative correlation between GA, BW, and CCT in premature infants and demonstrated that GA and BW could significantly predict the CCT values [11]. However, Karahan et al. showed that CCT did not correlate with GA or BW in their cross-sectional study [8]. Figure 3 in our study showed that the CCT of low BW (≤1500 g) was thicker than high BW (>1500 g). The difference in CCT was not apparent after 40 weeks. The same was true about GA. The CCT of low GA (≤30 weeks) was thicker than high GA (>30 weeks). The decrease in CCT reached an endpoint at 40 weeks and then kept relatively stable. In addition, presence of ROP did not contribute to change in CCT. In our study, gender, BW, and GA were found to be important influencing factors in change in CCT in premature infants. The reason of a different result might be due to a larger sample data and a longer follow-up period.

Kıvanç et al. conducted a study on comparing corneal thicknesses of prematurely born and full-term early school-aged children according to GA, and it was concluded that CCT in the small GA group was significantly lower than the appropriate GA group. In addition, CCT in the term children was higher than those of the prematurely born children [18]. In our study, premature infants were grouped as the thin CCT group (CCT < 566.66 *µ*m) and the thick CCT group (CCT ≥ 566.66 *µ*m) at 40 w. The two groups hold the similar decreasing rates, and the infants with thick CCT at 40 w also had a thicker CCT at 52 w and 64 w. This indicated that the CCT at 40 w had a correlation with that at one year, which could predict the CCT variation trend during the first year after birth.

This study has several limitations. The result of CCT value at the age of 2 to 3 years, or even older, was not included. The comparison of CCT in premature and full-term newborns or adults was not included in our study, which could be the future direction.

## 5. Conclusions

In our study, we found that the mean CCT had a weekly reduction at the early stage of life and then stabilized after 3 months of PMA, and the thick CCT at 40 w would maintain a thicker CCT at 52 w and 64 w, which could predict the variation trend of CCT during the first year after birth. The CCT measurements for each GA and PMA may contribute to a future reference parameter in diagnosing and managing pediatric glaucoma in premature infants.

## Figures and Tables

**Table 1:** Baseline characteristics of premature infants.

Characteristic	
Number of participants	1726
Male	943
Female	786
Total measurement times	3463
Mean BW (g)	1556.40 ± 409.63
Mean GA (w)	30.99 ± 2.22
Number of accompanying ROP	
Yes	362 (20.9%)
No	1364 (79.1%)

BW: birth weight; GA: gestational age; ROP: retinopathy of prematurity.

**Table 2:** Mean CCT at each PMA.

PMA	*N*	CCT (*μ*m) (mean ± SD)
34 w	524	602.67 ± 57.26
36 w	723	590.56 ± 55.06
38 w	585	581.72 ± 51.63
40 w	529	563.499 ± 51.63
42 w	293	551.72 ± 45.94
44 w	158	547.99 ± 47.81
46 w	148	538.91 ± 44.87
48 w	113	537.91 ± 37.09
52 w	150	529.17 ± 38.73
64 w	106	527.30 ± 41.24
76 w	69	527.20 ± 33.550
88 w	65	534.62 ± 37.01

PMA: postmenstrual age; *N*: number of infants; CCT: central corneal thickness.

**Table 3:** Mean CCT of different genders at each PMA.

PMA	Male CCT (mean ± SD)	*N*	Female CCT (mean ± SD)	*N*	*t*	*P*
34 w	611.13 ± 58.78	310	590.41 ± 52.76	214	4.13	＜0.001
36 w	601.35 ± 56.01	412	576.27 ± 50.41	311	6.22	＜0.001
38 w	588.86 ± 49.50	323	572.92 ± 52.92	262	3.75	＜0.001
40 w	572.86 ± 52.08	264	554.17 ± 49.54	265	4.23	＜0.001
42 w	557.88 ± 47.98	169	543.33 ± 41.75	124	2.71	0.01
44 w	554.32 ± 44.52	82	541.20 ± 45.85	76	1.83	0.07
46 w	541.40 ± 43.59	87	535.37 ± 46.78	61	0.80	0.42
48 w	539.32 ± 36.85	68	535.78 ± 37.78	45	0.49	0.62
52 w	537.18 ± 42.06	90	525.38 ± 33.07	60	1.83	0.07
64 w	532.49 ± 45.06	67	518.39 ± 32.29	39	1.71	0.09
76 w	529.29 ± 37.61	41	524.14 ± 26.87	28	0.62	0.54
88 w	531.59 ± 39.76	39	526.65 ± 35.79	26	0.51	0.61

PMA: postmenstrual age; *N*: number of infants; CCT: central corneal thickness.

**Table 4:** Mean CCT of different BWs at each PMA.

PMA	BW				*t*	*P*
	<1500 g		>1500 g			
	CCT (*μ*m)	*N*	CCT (*μ*m)	*N*		
34 w	619.10 ± 59.59	220	590.78 ± 52.49	304	5.76	<0.0001
36 w	605.28 ± 58.34	313	579.33 ± 49.63	410	6.45	<0.0001
38 w	591.01 ± 53.64	284	572.95 ± 48.12	301	4.29	<0.0001
40 w	574.83 ± 54.58	213	555.98 ± 48.21	318	4.18	<0.0001
42 w	554.42 ± 47.82	144	549.11 ± 44.05	149	0.99	0.32
44 w	551.55 ± 48.08	88	543.56 ± 41.94	70	1.09	0.27
46 w	542.00 ± 41.97	71	530.71 ± 45.97	77	1.56	0.12
48 w	540.37 ± 36.36	67	534.33 ± 38.26	46	0.85	0.40
52 w	538.00 ± 36.79	75	526.92 ± 40.64	75	1.75	0.08
64 w	530.50 ± 39.29	64	522.43 ± 44.07	42	0.99	0.33
76 w	531.56 ± 30.53	39	521.53 ± 36.85	30	1.24	0.22
88 w	534.215 ± 39.35	38	523.15 ± 35.75	27	1.16	0.25

PMA: postmenstrual age; *N*: number of infants; CCT: central corneal thickness; BW: birth weight.

**Table 5:** Mean CCT of different GAs at each PMA.

PMA	GA				*t*	*P*
	<30 w		>30 w			
	CCT (*μ*m)	*N*	CCT (*μ*m)	*N*		
34 w	619.67 ± 56.18	237	588.63 ± 54.35	287	6.41	<0.0001
36 w	605.33 ± 59.24	271	581.72 ± 50.43	452	5.70	<0.0001
38 w	589.96 ± 51.80	245	575.78 ± 50.75	340	3.31	0.001
40 w	572.54 ± 52.27	187	558.56 ± 50.67	342	3.00	0.003
42 w	554.22 ± 46.90	132	549.68 ± 45.18	161	0.84	0.40
44 w	552.40 ± 47.77	89	542.33 ± 42.05	69	1.38	0.17
46 w	541.08 ± 42.00	67	532.03 ± 45.99	81	1.24	0.22
48 w	540.03 ± 35.78	61	535.42 ± 38.78	52	0.66	0.51
52 w	535.11 ± 35.86	81	529.35 ± 42.50	69	0.13	0.37
64 w	533.56 ± 43.25	61	518.82 ± 37.15	45	1.84	0.07
76 w	531.93 ± 40.20	36	525.67 ± 34.41	33	0.18	0.85
88 w	539.85 ± 37.87	41	525.67 ± 34.41	24	0.25	0.53

PMA: postmenstrual age; *N*: number of infants; CCT: central corneal thickness; GA: gestational age.

**Table 6:** Mean CCT in the ROP subgroup and without the ROP subgroup at each PMA.

PMA	ROP				*t*	*P*
	Yes		No			
	CCT (*μ*m)	*N*	CCT (*μ*m)	*N*		
34 w	604.94 ± 52.08	100	602.13 ± 58.46	424	0.44	0.66
36 w	594.85 ± 57.45	154	589.40 ± 54.39	569	1.09	0.28
38 w	578.49 ± 50.49	144	582.78 ± 52.00	441	0.87	0.39
40 w	558.41 ± 44.18	123	565.04 ± 53.63	406	1.25	0.21
42 w	547.07 ± 45.19	81	553.50 ± 46.21	212	1.07	0.28
44 w	543.52 ± 48.84	68	551.37 ± 47.01	90	1.02	0.31
46 w	532.83 ± 44.67	57	540.33 ± 44.87	91	0.99	0.32
48 w	533.79 ± 36.86	58	542.26 ± 37.18	55	1.22	0.23
52 w	539.71 ± 41.11	66	526.76 ± 36.55	84	2.49	0.05
64 w	532.84 ± 44.58	57	520.86 ± 36.37	49	1.50	0.14
76 w	531.63 ± 38.24	30	523.80 ± 29.51	39	0.96	0.34
88 w	539.69 ± 32.11	32	529.70 ± 41.10	33	1.09	0.28

PMA: postmenstrual age; *N*: number of infants; CCT: central corneal thickness; ROP: retinopathy of prematurity.

**Table 7:** CCT variation trend at 40 w, 52 w, and 64 w of PMA.

PMA	CCT < 566.66 *µ*m (*N* = 36)	CCT ≥ 566.66 *µ*m (*N* = 29)	*t*	^*∗*^ *P*
40 w	527.72 ± 26.40	615.00 ± 34.36	−11.50	<0.001
52 w	508.53 ± 35.79	556.55 ± 40.77	−4.98	<0.001
64 w	506.58 ± 26.18	558.10 ± 44.01	−5.86	<0.001
*F*	5.536	20.189		
^†^ *P*	0.005	<0.001	—	

*F* represented the test statistic in one-way ANOVA. ^*∗*^*P* value represented CCT < 566.66 *µ*m vs CCT ≥ 566.66 *µ*m at each PMA; ^†^*P* value represented the CCT change trend at each PMA in CCT < 566.66 *µ*m and CCT ≥ 566.66 *µ*m.

## Data Availability

The data used to support the findings of this study are available from the corresponding author on request.
